# Effect of the SARS‐CoV‐2 pandemic on colorectal cancer diagnosis and prognosis

**DOI:** 10.1002/cam4.6923

**Published:** 2024-03-16

**Authors:** Lucía Medina‐Prado, Noelia Sala‐Miquel, Marta Aicart‐Ramos, Julia López‐Cardona, Marta Ponce‐Romero, Oswaldo Ortíz, María Pellisé, Lara Aguilera, Pilar Díez‐Redondo, Henar Núñez‐Rodríguez, Agustín Seoane, María‐José Domper‐Arnal, Cristina Borao‐Laguna, Óscar González‐Bernardo, Adolfo Suárez, María Muñoz‐Tornero, Marco Bustamante‐Balén, Carlos Soutullo‐Castiñeiras, Belén Balleste‐Peris, Pilar Esteban, Mirella Jiménez‐Gómez, Marc Albert, Javier Lucas, Eduardo Valdivieso‐Cortázar, Antonio López‐Serrano, Marina Solano, Javier Tejedor‐Tejada, Marita Trelles, Pedro Zapater, Rodrigo Jover

**Affiliations:** ^1^ Servicio de Medicina Digestiva, Hospital General Universitario Dr. Balmis, Instituto de Investigación Sanitaria ISABIAL, Departamento de Medicina Clínica Universidad Miguel Hernández Alicante Spain; ^2^ Hospital Ramón y Cajal Madrid Spain; ^3^ Hospital Clínico de Valencia Valencia Spain; ^4^ Hospital Clinic de Barcelona Barcelona Spain; ^5^ Hospital Vall d'Hebron. Gastroenterology department vall d'Hebron Research Institute Barcelona Spain; ^6^ Hospital Universitario Río Hortega Valladolid Spain; ^7^ Hospital del Mar Medical Research Institute (IMIM), Gastroenterology department Barcelona Spain; ^8^ Hospital Clínico Universitario Lozano Blesa. Instituto de investigación sanitaria de Aragón (IIS Aragón) Zaragoza Spain; ^9^ Hospital Universitario Central de Asturias Oviedo Spain; ^10^ Hospital Virgen de la Arrixaca El Palmar Spain; ^11^ Hospital La Fe Valencia Spain; ^12^ Hospital Joan XXIII Tarragona Spain; ^13^ Hospital Morales Meseguer. Instituto Murciano de Investigación Biosanitaria (IMIB) Murica Spain; ^14^ Hospital Universitario de la Princesa Madrid Spain; ^15^ Hospital Universitari de Girona Doctor Josep Trueta Girona Spain; ^16^ Hospital Universitario Fundación Alcorcón Alcorcon Spain; ^17^ Complejo Hospitalario Universitario A Coruña A Coruna Spain; ^18^ Hospital Universitario Doctor Peset Valencia Spain; ^19^ Hospital Comarcal de Alcañiz Teruel Spain; ^20^ Hospital Universitario de Cabueñes Gijon Spain; ^21^ Hospital Comarcal de Inca Mallorca Spain

**Keywords:** COVID‐19 pandemic, endoscopy, screening colonoscopy

## Abstract

**Background and Study Aims:**

Our aim was to determine the impact of the SARS‐CoV‐2 pandemic on the diagnosis and prognosis of colorectal cancer (CRC).

**Patients and Methods:**

This prospective cohort study included individuals diagnosed with CRC between March 13, 2019 and June 20, 2021 across 21 Spanish hospitals. Two time periods were compared: prepandemic (from March 13, 2019 to March 13, 2020) and pandemic (from March 14, 2020 to June 20, 2021, lockdown period and 1 year after lockdown).

**Results:**

We observed a 46.9% decrease in the number of CRC diagnoses (95% confidence interval (CI): 45.1%–48.7%) during the lockdown and 29.7% decrease (95% CI: 28.1%–31.4%) in the year after the lockdown. The proportion of patients diagnosed at stage I significantly decreased during the pandemic (21.7% vs. 19.0%; *p* = 0.025). Centers that applied universal preprocedure SARS‐CoV‐2 PCR testing experienced a higher reduction in the number of colonoscopies performed during the pandemic post‐lockdown (34.0% reduction; 95% CI: 33.6%–34.4% vs. 13.7; 95% CI: 13.4%–13.9%) and in the number of CRCs diagnosed (34.1% reduction; 95% CI: 31.4%–36.8% vs. 26.7%; 95% CI: 24.6%–28.8%). Curative treatment was received by 87.5% of patients diagnosed with rectal cancer prepandemic and 80.7% of patients during the pandemic post‐lockdown period (*p* = 0.002).

**Conclusions:**

The COVID‐19 pandemic has led to a decrease in the number of diagnosed CRC cases and in the proportion of stage I CRC. The reduction in the number of colonoscopies and CRC diagnoses was higher in centers that applied universal SARS‐CoV‐2 PCR screening before colonoscopy. In addition, the COVID‐19 pandemic has affected curative treatment of rectal cancers.

## 1 INTRODUCTION

Colorectal cancer (CRC) is the second most diagnosed cancer with an overall incidence of 93.8 per 100,000 inhabitants per year and caused 16,470 deaths in Spain in 2020.^1^

The SARS‐CoV‐2 pandemic led to a worldwide collapse of the healthcare system, requiring the redirection of material and human resources to the care of patients infected by the virus. In Spain, since the declaration of the state of alarm on March 14, 2020, scheduled healthcare activities were suspended following the recommendation of the main scientific societies related to digestive endoscopy, which agreed on the cessation of most nonurgent endoscopic activity, delaying colon cancer screening, post‐polypectomy surveillance, and direct screening of patients with a family history of CRC or hereditary syndromes.^2^, ^3^, ^4^ This decision led to an increase in the waiting lists and a delay in diagnosis of CRC, which could translate into fewer CRC diagnoses, more advanced stage at diagnosis, and a worst prognosis.^5^, ^6^ Robust evidence supports an association between delaying elective surgery >4 week in patients with CRC and poorer overall survival (OS) or disease‐free survival (DFS).^7^ Moreover, in patients with rectal cancer, delaying neo/adjuvant chemotherapy will lower their DFS and increase their risk of local and distant recurrence.^8^

Our aim was to determine the impact of the SARS‐CoV‐2 pandemic on the diagnosis, prognosis, and management of CRC in Spain by analyzing the differences in CRC staging and treatment received before and after the onset of the pandemic in Spain. Moreover, we have analyzed the effect of SARS‐CoV‐2 preprocedural PCR testing on the return to the normal colonoscopy activity during pandemic.

## 2 METHODS

This descriptive, prospective, multicenter, cohort study included individuals >18 years old diagnosed with CRC between March 13, 2019 and June 20, 2021 across 21 hospitals from different regions of Spain. We excluded patients diagnosed before March 13, 2019 and all the cases of colonic neoplasia other than adenocarcinoma of the colon or rectum.

We compared three time periods: a 1‐year prepandemic period reflecting the usual activity of the endoscopy units (52 weeks, from March 13, 2019 to March 13, 2020), a lockdown period that reflects the highest impact of the COVID‐19 pandemic on endoscopy activity established by the declaration of the state of alarm (lockdown period: 14 weeks, from March 14, 2020 to June 20, 2020), and a 1‐year postlockdown period with the transition and recovery of activity during the pandemic (52 weeks, from June 21, 2020 to June 21, 2021). Both lockdown and 1 year after lockdown are considered the pandemic period.

The register of patients diagnosed with CRC by biopsy and/or surgical specimen was obtained from the pathology departments of the different centers and included in the database anonymously by consecutive sampling. This study was approved by the Ethical Review Board of each participating center. This study was classified as public health research and, therefore, informed consent was not required for participants. The study protocol conformed to the ethical guidelines of the 1975 Declaration of Helsinki as reflected in a prior approval by the institution's Human Research Committee.

During the pandemic post‐lockdown period, some centers applied universal preprocedural SARS‐CoV‐2 PCR for all scheduled colonoscopies and some centers did not. In these last centers, all patients underwent screening for COVID‐19 symptoms prior to their endoscopy and PCR testing was performed only if patients show symptoms suggestive of SARS‐CoV‐2. We have analyzed differences between these centers in regard to the number of colonoscopies performed and number of CRC diagnoses in the prepandemic and pandemic post‐lockdown periods.

### 2.1 Definitions

The left colon includes sigma, descending colon, and splenic flexure; the transverse colon includes the hepatic flexure; and the right colon includes ascending colon, the cecum and ileocecal valve. Patient staging was determined according to the AJCC 8th edition guidelines.^9^ Treatment of curative intention was considered in patients who had undergone surgery for the primary neoplasm and, if appropriate, resective surgery for metastases, with or without neo/adjuvant chemotherapy or radiotherapy. Palliative intention was considered in patients who received only chemotherapy without surgery or symptomatic treatment.

### 2.2 Statistical analysis

Variables are expressed as the frequency (%) for categorical variables, the mean (standard deviation) for continuous variables, and the median with interquartile range (IQR: 25th‐75th) for discrete variables, depending on whether they followed a normal distribution. We performed univariate analyses with the chi‐squared test for categorical data and Mann–Whitney *U* test for non‐categorical variables, to compare differences in patient characteristics, tumor characteristics, staging, and treatment options in the set time periods. Values of *p* are two sided, and *p* < 0.05 was considered significant. We also report the 95% CI for proportions. All statistical analyses were performed with SPSS 25.0 software (IBM Corp., Armonk, NY, USA).

## 3 RESULTS

**FIGURE 1:**
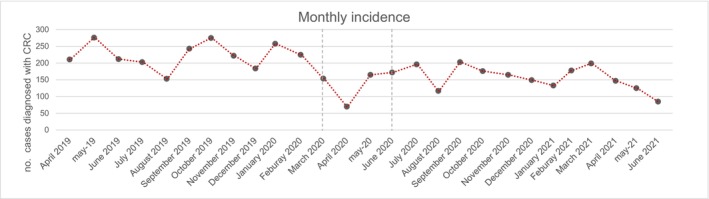
Monthly incidence of CRC in the prepandemic and pandemic periods.

Between March 2019 and June 2021, 5329 patients were diagnosed with CRC. Of these, 2886 were diagnosed in the prepandemic period and 2443 in the pandemic period, 415 during the lockdown and 2028 post‐lockdown. Thus, the number of CRC diagnoses decreased 46.9% during the lockdown and 29.7% in the year after the lockdown period compared to the prepandemic period. This decrease in CRC diagnoses runs parallel to the endoscopic activity, which decreased 55.0% during lockdown and 21.7% in the year after the lockdown (Figure 1). There were no significant changes in the CRC detection rate of these colonoscopies between the prepandemic and pandemic period: 2.3% (95% CI: 2.2%–2.4%) prepandemic, 2.7% (95% CI: 2.4%–3.0%) during lockdown, and 2.1% (95% CI: 2.0%–2.1%) during the pandemic post‐lockdown period (Table 1). The mean incidence was 240.5 cases per month in the prepandemic period, decreased to 127.7 cases per month during the lockdown period and 169.0 cases per month in the post‐lockdown period (Figure 1).

**TABLE 1:** CRC detection rate and missing CRC diagnoses in pre and postpandemic years and during lockdown.

	Prepandemic (13‐3‐19/12‐3‐20)	Pandemic (13‐3‐20/20‐6‐21)	Lockdown (13‐3‐20/20‐6‐21)	Pandemic post‐lockdown (21‐6‐20/20‐6‐21)	Total
Number of colonoscopies performed	125,964	113,993	15,357	98,636	239,957
Number of cancers detected	2886	2443	415	2028	5329
Average cancers detected per month	240.5	160.2	127.7	169.0	
CRC detection rate (95% CI)	2.3% (2.2–2.4)	2.1 (2.1–2.2)	2.7% (2.4–3.0)	2.1% (2.0–2.1)	
Reduction in number of colonoscopies (95% CI)		28.8% (28.5–29.0)	55.0% (54.7–55.3)	21.7% (21.5–21.9)	
Reduction in number of CRC diagnoses (95% CI)		33.4% (31.7–35.1)	46.9% (45.1–48.7)	29.7% (28.1–31.4)	

*Note*: Values are *n* (%).

### 3.1 Patient characteristics

Characteristics of patients at diagnosis are provided in Table 2. The mean age at diagnosis was 70.1 ± 12.7 years and 61% of cases were male. The most frequent location of CRC during both periods was the left colon (36%), followed by the right colon (26.6%) and the rectum (26.2%). Indications for colonoscopies varied between the prepandemic and pandemic periods. There was an increase in the proportion of patients diagnosed because of symptoms during the pandemic period (73.6% vs. 82.2%; *p* < 0.001) and a decrease in the proportion of patients diagnosed because of FIT‐based CRC screening (20.9% vs. 12.8%; *p* < 0.001). This decrease was more prominent during the lockdown (10.9%) but still persisted during the year post‐lockdown (13.2%).

**TABLE 2:** Characteristics of patients diagnosed with CRC. Values are *n* (%) or mean ± standard deviation.

Patient characteristics (*n*)	Prepandemic (13‐3‐19/13‐3‐20)	Lockdown (13‐3‐20/20‐6‐21)	Pandemic (13‐3‐20 /20‐6‐21)	Total	*p*‐value
Age	70.5 ± 12.8	69.9 ± 13.4	71.61 ± 2.5	71 ± 12.7	0.01
Sex (♂)	1766 (61.3)	260 (62.7)	1217 (60)	3243 (60.9)	0.52
Colonoscopy indication					<0.01
FOBTs + screening	569 (20.9)	42 (10.9)	253 (13.2)	864 (17.2)	
Symptoms	1999 (73.6)	332 (85.8)	1556 (81.4)	3887 (77.5)	
Imaging/physical examination alteration	486 (24.3)	111 (33.4)	490 (31.6)	1087 (28.0)	
Bleeding/change bowel rhythm	741 (37.2)	108 (32.5)	526 (33.9)	1375 (35.3)	
Iron deficiency anemia	620 (31.1)	95 (28,6)	439 (28.3)	1154 (29.7)	
Other	37 (2.0)	4 (1.2)	35 (2.0)	77 (2.0)	
Emergency: perforation/occlusion	177 (6.1)	29 (7.0)	135 (6.4)	341 (6.4)	
Surveillance	93 (3.4)	8 (2.1)	84 (4.4)	185 (3.7)	
Direct colonoscopy screening	56 (2.1)	5 (1.3)	18 (0.9)	79 (1.6)	
Neoplasia location					0.69
Rectum	744 (25.8)	105 (25.3)	546 (26.9)	1395 (26.2)	
Left colon	1039 (36)	165 (39.8)	714 (35.2)	1918 (36)	
Right colon	1101 (38.1)	145 (34.9)	768 (37.8)	2014 (37.8)	

*Note*: Values are *n* (%).

In symptomatic patients, during the prepandemic period, the most common symptoms that indicated a diagnostic colonoscopy were suspicion of CRC by diagnostic imaging or physical examination (24.3%), iron deficiency anemia (29.9%), and rectorrhagia/hematochezia (26.2%). These percentages changed during the pandemic period, with an increase in colonoscopies requested for suspected CRC by diagnostic imaging or physical examination (31.9%) and a decrease in studies for anemia (27.9%), rectorrhagia (23%), and other less frequent symptoms (Table 2). However, there were no differences between the prepandemic (6.1%) and pandemic periods (6.7%) in regard to the proportion of patients diagnosed because of complications of CRC requiring emergency surgery, such as perforation or occlusion.

### 3.2 Diagnosis trends and impact of preprocedural testing

**FIGURE 2:**
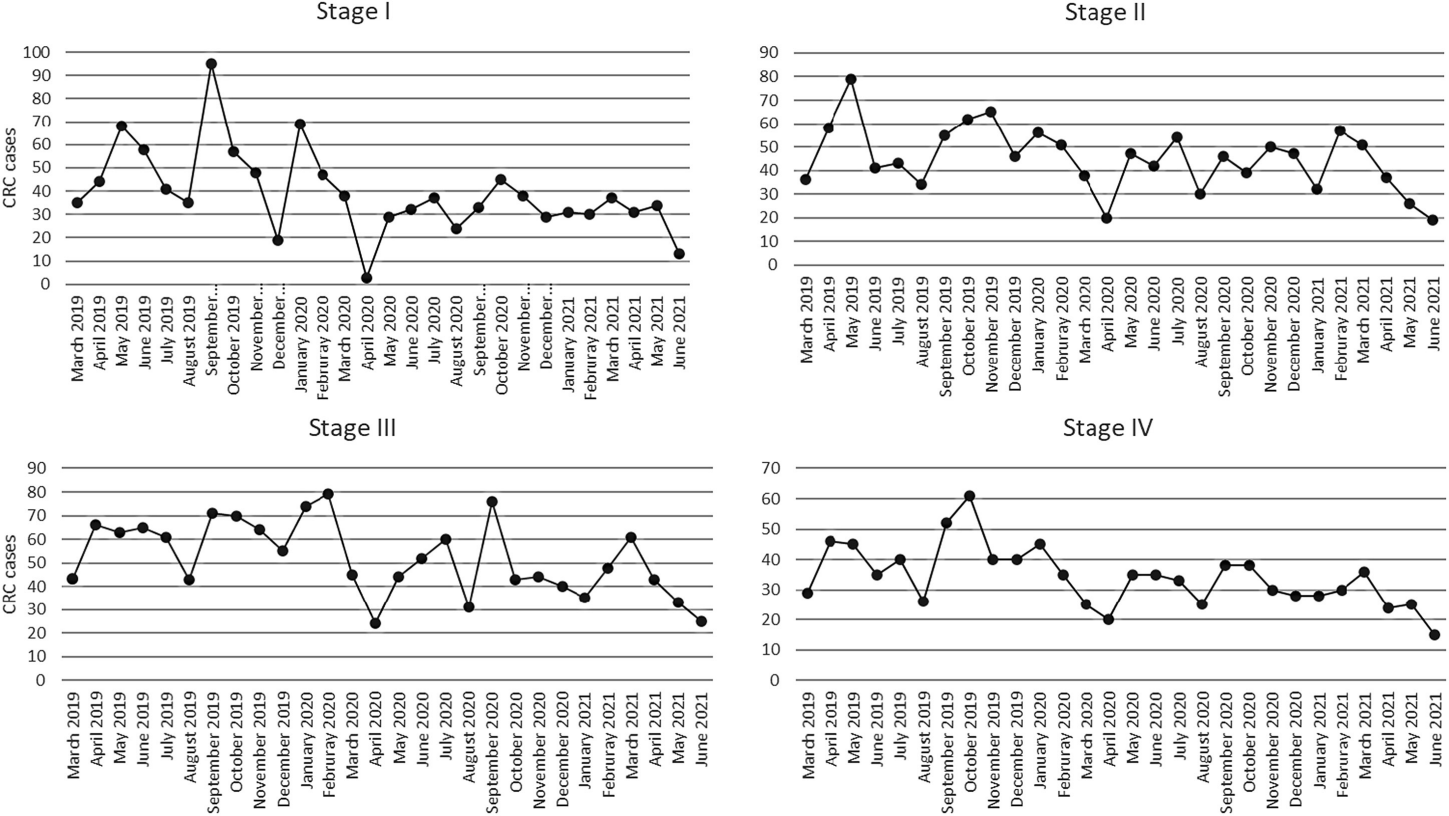
Diagnosis of CRC at different stages before and during the prepandemic and pandemic periods.

**FIGURE 3:**
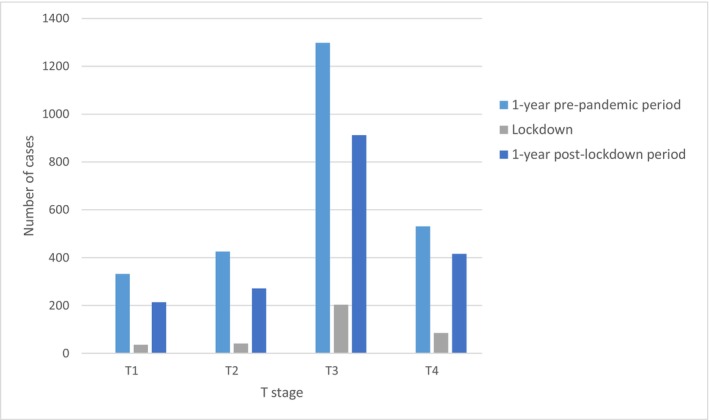
Diagnosis of the different *T* stages at different stages before and during the prepandemic and pandemic periods. The number of diagnoses was reduced during lockdown.

Regarding stage at diagnosis, the proportion of patients diagnosed at stage I significantly decreased during the pandemic period (21.7% vs. 19.0%; *p* = 0.025). On the other hand, there was a slight increase in the proportion of patients diagnosed at stage IV during the pandemic period (19.4% vs. 19.6%; *p* = 0.019). There were no differences in the proportion of patients diagnosed at stages II and III during both periods of the study (Table 3). Figure 2 shows the evolution of diagnoses in the different stages over the he months considered in the study. Figure 3 shows the *T* stage at the different phases of the pandemic.

**TABLE 3:** Stage of CRC during the different study periods.

CRC stage	Prepandemic (13‐3‐19/13‐3‐20)	Lockdown (13‐3‐20/20‐6‐21)	Pandemic (13‐3‐20/20‐6‐21)	*p*‐value
Stage I	639 (23.5)	64 (16.2)	404 (21.3)	0.003
Stage II	698 (24.4)	107 (25.8)	542 (26.7)	0.346
Stage III	827 (28.7)	129 (31.1)	579 (28.6)	0.133
Stage IV	559 (19.4)	95 (22.9)	385 (19.0)	0.012

*Note*: Values are *n* (%).

**FIGURE 4:**
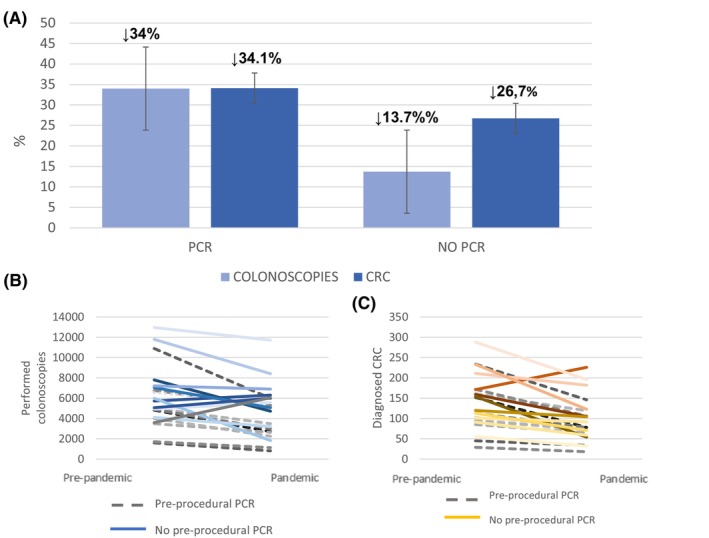
Effect of the SARS‐Cov‐2 PCR testing policy on the number of colonoscopies and diagnosis of CRC. (A) Percentage reduction in colonoscopies and CRC cases diagnosed during the pandemic post‐lockdown period compared to the prepandemic period in centers applying or not applying universal pre‐procedural SARS‐CoV‐2 PCR testing. (B) Total number of colonoscopies in individual centers with universal pre‐procedural PCR (dotted lines) or without universal pre‐procedural PCR (continuous lines). (C) Number of CRC cases diagnosed in individual centers with universal pre‐procedural PCR (dotted lines) or without universal pre‐procedural PCR (continuous lines).

During the pandemic period, 10 centers applied universal preprocedural testing before colonoscopies and 11 did not, only performing SARS‐CoV‐2 PCR testing in cases with clinical suspicion of COVID‐19. The centers that applied universal preprocedure PCR testing experienced a higher reduction in the number of colonoscopies performed during the pandemic post‐lockdown period compared to the prepandemic period (32,826 vs. 49,736 procedures; 34.0% reduction; 95% CI: 33.6%–34.4%) as well as in the number of CRC diagnoses during both periods (784 vs. 1189, respectively; 34.1% reduction; 95% CI: 31.4%–36.8%). On the other hand, in the centers that did not apply this policy, the reduction in the number of colonoscopies was smaller (prepandemic: 76,228; post‐lockdown: 65,810; 13.7% reduction; 95% CI: 13.4%–13.9%), as was the reduction in the number of CRC cases diagnosed (prepandemic: 1697; post‐lockdown: 1244; 26.7% reduction; 95% CI: 24.6%–28.8%). Figure 4A shows the reduction in the number of colonoscopies and CRC cases diagnosed depending on their management of preprocedure SARS‐CoV‐2 PCR testing. Figure 4B shows the variation in the number of colonoscopies, and Figure 4C shows the variation in the number CRC cases diagnosed at the different centers depending on preprocedure systematic or elective SARS‐CoV‐2 PCR.

### 3.3 Treatment patterns

Concerning treatment, a lower proportion of patients underwent surgery during the pandemic period (2296 patients, 79.6% vs. 1868, 76.5%; *p* < 0.001). If we differentiate between curative or palliative intention of treatment (Table 4), patients diagnosed with rectal cancer received curative treatment in 87.5% of cases during the prepandemic period and in 80.7% of cases during the pandemic period (*p* < 0.001). This situation remained similar during lockdown (20.0%) and in the post‐lockdown period (19.2%). However, no differences were observed in patients diagnosed with colon cancer.

**TABLE 4:** Differences in the type of treatment received for rectal and colon cancer.

Rectal cancer (*n* = 1392)	Prepandemic (13‐3‐19/13‐3‐20)	During lockdown (13‐3‐20/20‐6‐21)	Pandemic (13‐3‐20/20‐6‐21)	*p*‐value
Curative treatment	651 (87.5)	84 (80.0)	439 (80.8)	0.002
Palliative treatment	93 (12.5)	21 (20.0)	104 (19.2)	
Colon cancer (*n* = 3930)				
Curative treatment	1900 (88.8)	268 (86.5)	1300 (87.8)	0.407
Palliative treatment	240 (11.2)	42 (13.5)	180 (12.2)	

*Note*: Values are *n* (%).

## 4 DISCUSSION

In this study, we found that the COVID‐19 pandemic provoked an important decrease in the number of patients diagnosed with CRC, with a reduction in diagnosis of 47% during lockdown and almost 30% during the pandemic year post‐lockdown, with a trend that was maintained 1 year after the end of the lockdown period. This decrease in CRC diagnoses is due to a parallel decrease in endoscopic activity.^10^ The decrease in endoscopic activity and the number of CRC cases diagnosed was higher in centers that applied universal SARS‐CoV‐2 PCR preprocedural testing before colonoscopies. Moreover, we observed a decrease in the proportion of patients diagnosed in FIT‐based screening programs in Spain similar to what has happened in other countries worldwide.^11^ This led to a significant decrease in the proportion of patients diagnosed at Stage I. These results show the effect of the COVID‐19 pandemic on the health system, particularly on CRC. Given the relatively short period of lockdown, with an almost complete close of endoscopy units, the main effect of the pandemic has been due to the slow recovery to normal, with longer stoppage of screening.

Most studies have reported that the diagnosis of CRC has been significantly reduced during the pandemic^11^, ^12^, ^13^, ^14^, ^15^, ^16^ The majority of these studies analyzed the short‐term evolution of CRC diagnosis during and shortly after lockdown.^17^ In this study, we analyzed the shortfall in health activity during the lockdown period and also the slow recovery in diagnostic activity for CRC and the consequences on the stage at diagnosis and treatment of these patients. We can see how, 1 year after the end of the emergency status, there is still a 30% decrease in the number of diagnosed cases of CRC. The reasons for this maintained decrease in CRC diagnosis are the several barriers found for re‐establishing normal activity in endoscopy units and the subsequent waves of the pandemic that prevent the resumption of normal activity.^5^ Our study shows that the recovery of endoscopic activity has been especially delayed in centers that applied preprocedural universal SARS‐CoV‐2 PCR testing before colonoscopies, adding an additional barrier to endoscopy practice.

Our results in real life contradict some modeling studies^18^ that predicted an increase in the number of diagnosed cases quickly after the pandemic. Colonoscopies are the main bottleneck for CRC diagnoses. To improve the diagnostic capacity of colonoscopy across the health system, a 140% increase in monthly colonoscopy volumes is needed for a period of 3 months.^19^ This requires substantial and unlikely health system changes. For instance, there is an option for screening programs to extend the invitation interval, which has demonstrated no association with an increase in the interval CRC rate. The entire screening process was postponed only for a few months and individuals caught up their missed invitation.^20^ However, this kind of solutions have not been widely implemented and there are important differences between countries and regions on the success to restore the backlog provoked by the COVID‐19 pandemic.

Our data do not analyze the impact on mortality, and the effect of delayed presentation on patients with cancer is not immediate. However, there are several worrisome findings that can provoke a further impact on mortality. On the one hand, the decrease in the number of patients diagnosed reveals a potential population of undiagnosed people, probably secondary to the stop of the screening programs. On the other hand, the reduction in the proportion of stage I patients also reveals that these patients will be diagnosed in the future in more advanced stages.^21^, ^22^ Modeling studies predict an excess of CRC related deaths in future years, that could increase even more if recovery periods are longer.^23^

Finally, the reduction in rectal cancer patients treated with curative intention could lead to worst prognosis, as seen in some modeling studies.^24^ Other factors may have influenced this change in the treatment of rectal cancer patients other than the delayed diagnosis of cancer. In addition, the surgical delay could compromise resectability and, in the case of rectal cancer, the possibility of neoadjuvant radiotherapeutic treatment.^7^, ^8^, ^25^ Previous data revealed higher therapeutic delay in rectal cancer than in colon cancer.^26^

Previous studies showed different situations regarding the recovery of activity after the lockdown phase of the SARS‐CoV‐2 pandemic. In some places, the activity came back to almost normal more quickly than in others.^17^, ^18^ However, this is probably too soon to gain a complete picture of the healthcare situation after this unprecedented situation. In this study, we found a significant decrease in the proportion of stage I CRC cases, something that has already been described^27^ and can be attributable to the stoppage of CRC screening programs. In contrast to this study, we did not observe a rapid catch up in the number of CRC cases diagnosed. There are several reasons for this: the unequal restart of CRC screening in the different Spanish regions; the reluctance of patients to participate in these screening programs after a positive FIT result; further challenges faced by endoscopy services in increasing capacity back to prepandemic levels, such as staff absences, infection control measures and additional administrative burden such as telephone triaging and preprocedural SARS‐CoV‐2 testing.^28^ These situations can conduct an inequities on the resumption of screening. Some studies have suggested that the COVID‐19 pandemic can have an uneven effect on CRC outcomes depending on whether and how fast screening is resumed after the pandemic onset.^29^

The strengths of our study are the ascertainment of cases one by one at the participating centers, in contrast to registry studies, for which information could be insufficient. In addition, the prospective nature of the data, at least in the post‐lockdown period. Despite the multicenter nature of the data, representative of the whole country and of secondary and tertiary hospitals. There are also some limitations as its retrospective nature, the lack of long‐term data that allows the analysis of mortality and the need for a larger sample size, which could allow better discrimination of the effect of delays on CRC stage at diagnosis.

In summary, our research shows the enormous strain the COVID‐19 pandemic has put on CRC diagnosis and the treatment of CRC patients, with a decrease in the number of CRC cases diagnosed due to the COVID‐19 pandemic that has not been caught up 1 year after the end of the emergency status. This decrease was mainly due to a reduction in stage I CRC cases, related to the reduction in CRC screening programs. Moreover, although other factors related to the patient and the type of tumor may also be involved, the pandemic has affected curative treatment of rectal cancers, probably due to delays in neoadjuvants chemotherapy and radiaton therapy.^11^ Strategies to increase endoscopy and CRC screening program capacity must be implemented in order to recover the backlog caused by the COVID‐19 pandemic.

Our data demonstrate the deleterious role of universal preprocedural SARS‐CoV‐2 PCR testing in the recovery of the endoscopic capacity, a practice that may be unnecessarily delaying procedures and diverting resources to activities with marginal, if any, benefits^30^ and with contradictory recommendations from different scientific societies.^4^, ^31^ The potential effect of this impairment in CRC diagnosis and treatment on mortality should be monitored prospectively.

## AUTHOR CONTRIBUTIONS

**Lucía Medina‐Prado:** Data curation (equal); formal analysis (equal); investigation (equal); methodology (equal); project administration (equal); writing – original draft (equal); writing – review and editing (equal). **Noelia Sala‐Miquel:** Data curation (equal); formal analysis (equal). **Marta Aicart‐Ramos:** Data curation (equal). **Julia López‐Cardona:** Data curation (equal). **Marta Ponce Romero:** Data curation (equal). **Oswaldo Ortíz:** Data curation (equal). **María Pellisé:** Conceptualization (equal); data curation (equal); visualization (equal). **Lara Aguilera:** Data curation (equal). **Pilar Díez Redondo:** Data curation (equal); validation (equal); visualization (equal). **Henar Núñez‐Rodríguez:** Data curation (equal); visualization (equal). **Agustín Seoane:** Data curation (equal); validation (equal); visualization (equal). **María‐José Domper‐Arnal:** Data curation (equal); validation (equal). **Cristina Borao‐Laguna:** Data curation (equal). **Óscar González‐Bernardo:** Data curation (equal); visualization (equal). **Adolfo Suárez:** Data curation (equal); validation (equal); visualization (equal). **María Muñoz‐Tornero:** Data curation (equal); visualization (equal). **Marco Bustamante‐Balén:** Data curation (equal); validation (equal); visualization (equal). **Carlos Soutullo Castiñeiras:** Data curation (equal); visualization (equal). **Belén Balleste‐Peris:** Data curation (equal); visualization (equal). **Pilar Esteban:** Data curation (equal); visualization (equal). **Mirella Jiménez Gómez:** Data curation (equal); visualization (equal). **Marc Albert:** Data curation (equal); validation (equal); visualization (equal). **Javier Lucas:** Data curation (equal); visualization (equal). **Eduardo Valdivieso Cortázar:** Data curation (equal); visualization (equal). **Antonio López‐Serrano:** Data curation (equal); visualization (equal). **Marina Solano:** Data curation (equal); visualization (equal). **Javier Tejedor‐Tejada:** Data curation (equal); visualization (equal). **Marita Trelles:** Data curation (equal); visualization (equal). **Pedro Zapater:** Conceptualization (equal); data curation (equal); formal analysis (equal); methodology (equal); validation (equal); visualization (equal). **Rodrigo Jover:** Conceptualization (equal); formal analysis (equal); funding acquisition (equal); investigation (equal); methodology (equal); project administration (equal); supervision (equal); validation (equal); visualization (equal); writing – review and editing (equal).

## CONFLICT OF INTEREST STATEMENT

The authors declare no competing interests.

## FUNDING INFORMATION

This work was supported by ISABIAL UPG‐20‐096 grant and Instituto de Salud Carlos III (PI20/01527), Asociación para la Investigación en Gastroenterología de la Provincia de Alicante (AIGPA), a private association that promotes research in gastrointestinal diseases in Alicante, supported the logistical aspects of the study. This association declare no conflicts of interest.

## Data Availability

All data supporting the conclusions of this study are available in the article and in the supplementary information (Tables 1, 2, 3, 4 and Figures 1, 2, 3, 4). Information on the identities of the patients is only contained in an anonymised database and is not shown in this article due to confidentiality law.

## APPENDIX A CONTRIBUTORS

### A.1

**Hospital General Universitario Dr. Balmis:** Lucía Medina‐Prado, Noelia Sala‐Miquel, Pedro Zapater, Sandra Baile‐Maxía, Carolina Mangas‐Sanjuan, José R. Aparicio, Juan Martínez‐Sempere, Francisco A. Ruíz‐Gómez, Belén Martínez‐Moreno, Luis Compañy, Lucía Guilabert, Rodrigo Jover. **Hospital Ramón y Cajal:** Sofía Parejo Carbonell, Beatriz Peñas García, Luis Cristian Perna Monroy. **Hospital Clínico de Valencia**: Carolina Martínez. **Hospital Clinic de Barcelona**: Gloria Ramoneda, Miriam Cuatrecas, Karmele Saez de Gordoa, María Daca. **Hospital Vall d'Hebron**: Elena Céspedes Martínez. **Hospital Río hortega:** Laura Sánchez Delgado, Carlos Maroto Martín. **Hospital del Mar:** Luis Barranco Priego, Faust Riu Pons, Miguel Pantaleón Sánchez, Josep María Dedeu Cusco, Bouchra Alouali Moussakhkhar, Rocío Pérez Berbegal. **Hospital Virgen de la Arrixaca:** Akiko Ono. **Hospital Morales Meseguer:** Laura Molina Faura. **Hospital Josep Trueta:** Virginia Piñol Sánchez. **Complejo Hospitalario Universitario A Coruña:** Pedro Alonso Aguirre, Lidia González Peñas. **Hospital Universitario Doctor Peset:** Rafael Díaz Muñoz. **Hospital Comarcal de Inca:** José Reyes Moreno, Alexandra Gene Hey.

## APPENDIX B AUTHOR CONTRIBUTION

### B.1

This manuscript is the result of a cooperative study conducted in 21 hospitals from different regions of Spain. Therefore, different collaborators have participated in the data collection which makes the number of authors somewhat higher than usual. All authors meet the authorship requirements. Unfortunately, we cannot remove any of the participant authors. Rodrigo Jover, created and designed the project. Lucía Medina‐Prado and Noelia Sala‐Miquel1, Pedro Zapater and Rodrigo Jover contributed with the analysis and interpretation of the data. Pedro Zapater provided methodological and statistical support. Lucía Medina‐Prado and Rodrigo Jover drafted the article. Lucía Medina‐Prado, Noelia Sala‐Miquel, Marta Aicart‐Ramos, Julia López‐Cardona, Marta Ponce Romero, Oswaldo Ortíz, María Pellisé, Lara Aguilera, Pilar Díez Redondo, Henar Núñez‐Rodríguez, Agustín Seoane, María‐José Domper‐Arnal, Cristina Borao‐Laguna, Óscar González‐Bernardo, Adolfo Suárez, María Muñoz‐Tornero, Marco Bustamante‐Balén, Carlos Soutullo Castiñeiras, Belén Balleste‐Peris, Pilar Esteban, Mirella Jiménez Gómez, Marc Albert, Javier Lucas, Eduardo Valdivieso Cortázar, Antonio López‐Serrano, Marina Solano, Javier Tejedor‐Tejada, Marita Trelles led the project from their hospitals and were involved in the selection of patients to be included, investigation of medical records and data collection in the anonymised database. The contributors shown in appendix 1 have worked on the database. All authors have contributed to critical revision of the article and final approval.
